# Acupuncture for uterine fibroids: Protocol for a systematic review of randomized controlled trials

**DOI:** 10.1097/MD.0000000000014631

**Published:** 2019-02-22

**Authors:** Tong Liu, Jiani Yu, Weichuan Kuang, Xiaoyin Wang, Jiang Ye, Xiaojia Qiu, Wen Xi, Yao Zeng, Hanhong Zou, Yue Liu

**Affiliations:** a Department of Acupuncture and Rehabilitation, GuangDong Second Hospital of Traditional Chinese Medicine; b Department of Rehabilitation Medicine, Guangdong Provincial Hospital of Traditional Chinese Medicine, Guangzhou, Guangdong, China.

**Keywords:** acupuncture, protocol, systematic review, uterine fibroids

## Abstract

**Background:**

Uterine fibroids represent the most common gynecological benign tumors in reproductive females. Acupuncture has been applied as a therapeutic modality in China to treat uterine fibroids. However, currently, few critical systematic reviews regarding the effect of acupuncture on uterine fibroids have been published. Our proposed review aims to evaluate the current evidence on the efficacy of acupuncture for uterine fibroids.

**Methods:**

A total of 7 databases were searched from their inception to December 2018, including PubMed, Medline, Embase, the Cochrane Central Register of Controlled Trials, the Chinese National Knowledge Infrastructure database, the Chinese Biomedical database, and the Wanfang database. The primary outcomes will be reduction in uterine volume and number of fibroids. Secondary outcomes are pelvic or low-back pain, assessed by Visual Analog Scale (VAS); Irregular menstrual periods; Low-abdominal pressure symptoms such as frequent or urgent urination, or constipation and adverse events. Data synthesis will be computed by RevManV.5.3.5 software when a data-analysis is allowed. Methodological quality will be evaluated with the risk of bias according to Cochrane Handbook.

**Results:**

This study will provide high-quality evidence of acupuncture for uterine fibroids.

**Conclusion:**

The conclusion of this systematic review will provide evidence to judge whether acupuncture is an effective therapeutic intervention for patients with uterine fibroids.

**Trial registration number:**

PROSPERO CRD42019120484.

## 1 Introduction

Uterine fibroids, also known as leiomyomas, represent the most common gynecological benign tumors in reproductive females, with pathogenesis remains largely unknown. The estimated incidence varied from 20% to 40% in reproductive years^[1,2]^ while nearly 70% at age 50.^[3]^ The related symptoms are always heavy and prolonged uterine bleeding, pelvic pain, and reproductive dysfunction,^[4]^ which significantly decreased patient's quality of life. In addition, study reported that pregnant women with uterine fibroids may take risk for cesarean delivery, breech presentation, and postpartum hemorrhage.^[5]^

Majority of treatment options have been applied for uterine fibroids, such as oral progestogens,^[6]^ surgery,^[7]^ and latest high intensity focused ultrasound (HIFU).^[8]^ However, all the treatments are associated with substantial side effects or risks. Acupuncture has been widely applied as a therapeutic modality in China and abroad, however, its effect for uterine fibroids has not been determined. Herein, we conducted this review to evaluate the effect of acupuncture for uterine fibroids.

## 2 Methods

Our review protocol has been registered on PROSPERO with number CRD42019120484 (https://www.crd.york.ac.uk/PROSPERO/display_record.php?RecordID=120484). Cochrane Handbook for Systematic Reviews of Interventions and the Preferred Reporting Items for Systematic Reviews and Meta-Analysis Protocol (PRISMA-P) statement guidelines strictly comply in the protocol.^[9]^ Any change of the review will be described if needed.

### 2.1 Inclusion criteria for study selection

#### 2.1.1 Types of studies

All clinical randomized controlled trials (RCTs) that label acupuncture for uterine fibroids are included without any language or publication status restrictions. Animal studies and duplication of published papers are excluded. Quasi-randomized trials and trials that we cannot ascertain if the trial is truly randomized are also excluded.

#### 2.1.2 Types of participants

Women of reproductive age with a diagnosis of uterine fibroids will be enrolled in the review without considering any information related to their age, sex, race, education, and nationality. The diagnosis could be confirmed by surgery, ultrasound, clinical signs, and symptoms.

#### 2.1.3 Types of interventions

##### 2.1.3.1 Experimental interventions

The types of acupuncture included traditional acupuncture, electroacupuncture, auricular acupuncture, scalp acupuncture, superficial acupuncture, wrist-ankle acupuncture, fire needling, warm needling, and abdominal acupuncture. Nonpenetrating point stimulation such as acupressure, transcutaneous electrical nerve stimulation, laser acupuncture is excluded. In addition, there is no limitation to the treatment cycle and frequency.

##### 2.1.3.2 Control interventions

The controls could be sham acupuncture, placebo acupuncture, acupressure, no treatment, non-acupoint acupuncture, medication, massage. Studies that compared acupuncture plus another therapy with the same other therapy alone will also be included. Trials that only involve comparisons between different types of acupuncture are excluded.

#### 2.1.4 Types of outcome measures

##### 2.1.4.1 Primary outcomes

(1) Reduction in uterine volume;
(2) Reduction in number of fibroids.

##### 2.1.4.2 Secondary outcomes

(1) Pelvic or low-back pain, assessed Visual Analog Scale (VAS);
(2) Irregular menstrual periods;
(3) Low-abdominal pressure symptoms such as frequent or urgent urination, or constipation;
(4) Adverse events.

### 2.2 Search methods for the identification of studies

#### 2.2.1 Electronic searches

The research team searched the following databases from their inception to December 2018, with no language restrictions: PubMed, Medline, Embase, the Cochrane Central Register of Controlled Trials, the Chinese National Knowledge Infrastructure database, the Chinese Biomedical database, and the Wanfang database. Keywords were acupuncture, electroacupuncture, needling, acupoint combined with uterine fibroids, myomas, leiomyomas, leiomyomatas, Pin Yin (including “Zhen Ci, or Zhen Jiu, or Hao Zhen, or Ti Zhen, or Dian Zhen, or Tou Zhen, or Fu Zhen, or Shou Zhen, or Huo Zhen, or Wanhuai Zhen, or Wen Zhen” and “Zi Gong Ji Liu, or Ji Liu” in the title and keywords sections).

#### 2.2.2 Searching other resources

Clinical trial registries, dissertations, and grey literature will be additionally searched. Furthermore, the reference lists of selected studies will also be scanned for additional studies. We will also search WHO International Clinical Trials Registry Platform (ICTRP) (http://apps.who.int/trialsearch/), ClinicalTrials.gov registry (http://clinicaltrials.gov/), Chinese Clinical Trial Registry and other relevant trial registries.

### 2.3 Data collection and analysis

#### 2.3.1 Selection of studies

**Figure 1:**
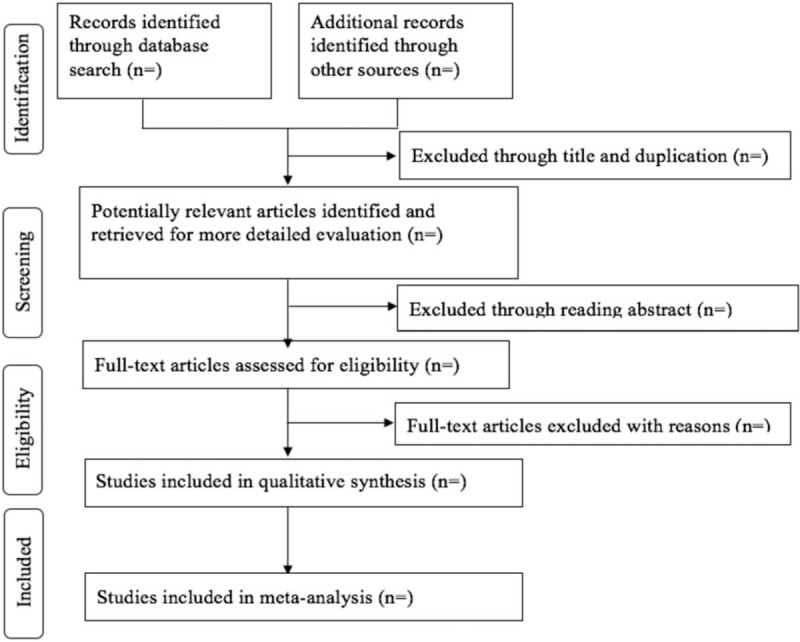
Flow diagram of study selection process.

Before selection of studies, all reviewers will receive professional training to understand the objective and process of the review. EndNote X8 software will be applied to upload the studies obtained from electronic databases and other resources. The selection of studies for the current review was performed independently by 2 reviewers (LT and YJN) screening the titles, abstracts, and keywords of all retrieved records separately. Any disagreement will be resolved through discussion and adjudication to get a consensus and judged by an arbiter (LY). A table named “reasons for excluded studies” will be established for the excluded studies. Details of the selection procedure for studies are shown in a PRISMA flow chart (Fig. 1).

#### 2.3.2 Data extraction and management

Data will be independently extracted from the selected studies and fill in the data collection form by 2 reviewers (LT and YJN). The following key information was extracted from each study:

(1) name of the first author,
(2) publication year,
(3) sample size,
(4) characteristics of the participants,
(5) intervention and control treatments,
(6) main acupoints or sites selected,
(7) duration and number of sessions of treatment,
(8) outcome measures, and
(9) any adverse events.

Any disagreement regarding the data extraction will be discussed and judged between the 2 authors or consulting a senior reviewer (LY). The results of the data extraction will be checked by the arbiter. When the data of articles are sufficient or ambiguous, we will contact the corresponding authors for more information by e-mail or telephone.

#### 2.3.3 Assessment of risk of bias in included studies

The research team conducted a risk-of-bias assessment using the Cochrane Collaboration's tool for assessing risk,^[10]^ which includes 6 dimensions:

(1) adequate sequence generation,
(2) allocation concealment,
(3) blinding,
(4) the presence of incomplete data,
(5) selective reporting, and
(6) other forms of bias.

The research team completed the risk-of-bias assessment for each study independently, with any discrepancies resolved through discussion. The quality of each trial was categorized into 1 of 3 types of risk of bias:

(1) low (L),
(2) unclear (U), and
(3) high (H).

Trials were categorized as having:

(1) a low risk of bias if all of the items had a low risk of bias,
(2) a high risk of bias if at least 1 item had a high risk of bias, and
(3) an unclear risk of bias if at least 1 item was unclear.

#### 2.3.4 Measures of treatment effect

Outcome data were summarized using a risk ratio (RR) with 95% confidence intervals (CIs) for binary outcomes or mean differences (MDs), with a 95% CI for continuous outcomes.

#### 2.3.5 Unit of analysis issue

Considering that some studies compared 2 or more intervention groups with a control group, the research team followed the recommended advice in the Cochrane Handbook version 5.1.017 and combined groups to create a single pairwise comparison to avoid a unit-of-analysis error.

#### 2.3.6 Dealing with missing data

We will try to contact the first author by e-mail or telephone to obtain the missing data if possible. If failed, we will analyze the available data and discuss the potential influence of the missing data in the discussion.

#### 2.3.7 Assessment of heterogeneity

Heterogeneity will be assessed with a standard x2 text according to the guideline of Cochrane Handbook. When the I^2^ value is <50%, Study will be regarded as no statistical heterogeneity if I^2^ value is <50%, and the fixed-effect model will be selected. It will be considered significant heterogeneity while I^2^ ≥50%, and we will select a random-effect model and make subgroup analysis to explore the potential causes of heterogeneity.

#### 2.3.8 Data synthesis and analysis

Review Manager Software (RevMan V.5.3.5) from Cochrane Collaboration will be applied for data synthesis and analysis. A random-effects model will be used when I^2^ ≥50% while a fixed-effects model will be applied when I^2^ <50%. When significant clinical heterogeneity existed, we will use subgroup analysis or sensitivity analysis, or only descriptive analysis.

#### 2.3.9 Assessment of publication bias

A funnel plot analysis was conducted to determine publication bias if 10 or more studies are in the meta-analysis.

#### 2.3.10 Subgroup analysis

Subgroup analyses were performed for type of acupuncture if at least 2 trials were available for which I^2^ >50%.

#### 2.3.11 Sensitivity analysis

Sensitivity analysis will be performed to identify the robustness of studies according to the following criteria: methodological quality, sample size, and missing data.

#### 2.3.12 Grading the quality of evidence

Grading of Recommendations Assessment, Development, and Evaluation (GRADE) will be applied to evaluate the quality of confidence for primary outcomes in including studies.^[11]^ The evaluation will divide into 4 levers: high, moderate, low, or very low.

## 3 Discussion

Uterine fibroids are common in reproductive women worldwide. Although mostly asymptomatic, it could be associated with a variety of healthy problems.^[12]^ A recent study highlights that nearly 50% of women in the USA rely on complementary and alternative medicine, including exercise (45%), diet (34%), herbs (37%), and acupuncture (16%), to treat symptoms of UFs. To be excited, patients using these interventions report significant symptomatic improvement than those using pharmacological and surgical treatments.^[13]^

Acupuncture has been widely studied to treat gynecological conditions.^[14]^ In our previous study, we also found that acupuncture may have potential effect for primary dysmenorrhea.^[15]^ Although a previous Cochrane review had assessed the benefits and harms of acupuncture in women with uterine fibroids in 2010, however, no trial was included in their review finally, therefore no data was collected.^[16]^ Since there have been majority of new RCTs involving acupuncture for uterine fibroids after 2010, it is, therefore, necessary to make a new review. We hope that this systematic review will help clinicians make decisions in practice and promote the progress of acupuncture research.

However, there are limitations that may affect the conclusion. First, different acupuncture types or acupoints may result in significant heterogeneity. Second, different age of patients and degree of uterine fibroids may also run risk of heterogeneity.

## Author contributions

Tong Liu and Jiani Yu contributed to the conception of the study. The manuscript of the protocol was drafted by Tong Liu and was revised by Jiani Yu and Yue Liu. The search strategy was developed by Weichuan Kuang, Xiaoyin Wang, and run by Yao Zeng and Hanhong Zou, who will also independently screen the potential studies, extract data of included studies, assess the risk of bias and finish data synthesis. Tong Liu and Xiaojia Qiu will arbitrate the disagreements and ensure that no errors occur during the study. All authors have approved the publication of the protocol.

**Conceptualization:** Tong Liu, Jiani Yu.

**Data curation:** Tong Liu, Jia-Ni Yu, Weichuan Kuang, Xiaoyin Wang, Yao Zeng, Hanhong Zou.

**Formal analysis:** Jiang Ye, Xiaojia Qiu, Wen Xi.

**Funding acquisition:** Yue Liu.

**Resources:** Tong Liu, Jia-Ni Yu.

**Writing – original draft:** Tong Liu, Jia-Ni Yu.
